# Association of Dietary Carrot Intake With Bladder Cancer Risk in a Prospective Cohort of 99,650 Individuals With 12.5 Years of Follow-Up

**DOI:** 10.3389/fnut.2021.669630

**Published:** 2021-07-26

**Authors:** Xin Xu, Yi Zhu, Sunyi Ye, Shiqi Li, Bo Xie, Hongzhou Meng, Shuo Wang, Dan Xia

**Affiliations:** Department of Urology, The First Affiliated Hospital, School of Medicine, Zhejiang University, Zhejiang, China

**Keywords:** carrot, carotene, bladder cancer, cohort, PLCO

## Abstract

Previous studies have provided limited evidence for the effect of carrot intake on bladder cancer incidence. This study aimed to evaluate the association between carrot consumption and bladder cancer risk in the Prostate, Lung, Colorectal, and Ovarian Cancer (PLCO) Screening cohort. PLCO enrolled 154,897 participants between November 1993 and July 2001 from 10 clinical screening centers throughout the United States. Hazard ratios (HRs) and 95% confidence intervals (CIs) were estimated using Cox regression model adjusting for confounders. A meta-analysis was also performed based on all available prospective studies with DerSimonian and Laird random-effects model to calculate summary relative risk (RR) and 95% CI. After a median of 12.5 years of follow-up, 762 incident bladder cancer cases occurred. We found no statistically significant association between dietary carrot intake and bladder cancer risk. The multivariate-adjusted HR of bladder cancer for participants in the highest category of total carrot intake compared with those in the lowest category was 0.96 (95% CI: 0.76–1.22; *P* for trend = 0.436). Corresponding adjusted HR was 0.98 (95% CI 0.90–1.06) per 1 SD increment of carrot intake. A meta-analysis based on two previous cohort studies and our study also found no significant association between carrot intake and bladder cancer risk (Summary HR 1.02, 95% CI 0.95–1.10) without obvious heterogeneity between studies (*P* = 0.859, *I*^2^ = 0.0%). In summary, analysis of the PLCO cohort did not provide evidence that dietary consumption of carrot was associated with the risk of bladder cancer.

## Introduction

Bladder cancer is the ninth most common cancer worldwide and is responsible for 430,000 cancer cases per year (1). Approximately 75% of patients present with non-muscle-invasive disease, whereas the remaining 25% have muscle-invasive disease (2). Despite the significant progress that has been made recently in immunotherapy, there remains an urgent need to improve bladder cancer prevention and outcomes (3, 4). Smoking is the most important risk factor for bladder cancer with an attributable risk of ~50% (5). Occupational carcinogen exposure amounts to 5–6% of the attributable-risk of bladder cancer (6). Less-established risk factors included such as lack of physical activity (7) and high intake of processed red meat (8).

Carrot has high amounts of α-carotene and β-carotene and may contribute to cancer prevention (9). Carotenoid was found to be able to inhibit oxidative damage to DNA at low concentrations and have been hypothesized to be anticancer agents (10, 11). It has been shown that β-carotene upregulates the PPAR-γ-mediated expression of antioxidant enzymes (12, 13). Several epidemiological studies have shown a potential inverse association between dietary carrot intake and bladder cancer risk (14–16) and a meta-analysis reported that carrot consumption was significantly associated with a decreased risk of bladder cancer (17). However, only two prospective studies (18, 19) were eligible in this meta-analysis with inconsistent results. To contribute to the conflicting and limited evidence base, we examined the association between dietary carrot consumption and bladder cancer risk in the Prostate, Lung, Colorectal and Ovarian (PLCO) cohort. The role of α-carotene and β-carotene intake in bladder cancer risk was also not completely clear and thus we further investigated the potential relationship between consumption of α-carotene or β-carotene and bladder cancer risk in the PLCO cohort.

## Methods

### Subjects and Study Design

The study design and methods of Prostate, Lung, Colorectal and Ovarian (PLCO) screening trial have been previously described (20). Briefly, the PLCO study is a population-based clinical trial aimed to determine whether certain screening tests would reduce death from prostate, lung, colorectal, and ovarian cancer. PLCO consisted of 154,897 eligible participants and enrolled between November 1993 and July 2001. The participants were from 10 clinical screening centers throughout the United States. Each of the 10 screening centers developed a recruitment plan. Various methods were used, including direct mail, community outreach, and mass media. PLCO participants were younger (within the 55–74 age range), were less racially diverse, and had higher education level than the 2000 US population of that age. In addition, they were more likely to be white and married (21). PLCO study was approved by the institutional review boards of the National Cancer Institute (NCI) and each of the participating centers. Informed consent was obtained from each eligible participant in the study. Our study was approved by the NCI with the number of PLCO-446.

### Data Collection and Dietary Assessment

The baseline questionnaire included self-reported information on demographic information, medical history, health behaviors and other factors. Dietary data were collected using the Diet History Questionnaire (DHQ) version 1.0, which queried frequency and portion size intakes of 124 individual food items and supplement use over the previous year (22). The DHQ has been validated to be as good as or better than two widely used food frequency questionnaires (FFQs) at the time the PLCO study was carried out (22). The USDA 1994 to 1996 Continuing Survey of Food Intakes by Individuals (23) were used to calibrate DHQ data and calculate the daily intake of carrots, α-carotene and β-carotene. The DHQ has been validated against 24-h dietary recalls (one in each season) among 1,640 nationally representative participants in the Eating at America's Table Study (22).

### Subject Selection

Individuals were excluded from this study if they did not return a baseline questionnaire (*n* = 4,920); did not complete DHQ or the DHQ was not valid (*n* = 48,256); participants with extreme energy (i.e., lowest or highest 1%) intakes (*n* = 2,033); had died of an unknown cause or had an undetermined case status (*n* = 37). Therefore, the cohort for analysis consisted of 99,650 participants.

### Outcome Assessment

Participants were followed until cancer diagnosis or death, or end of follow-up (December 31, 2009). Study participants were mailed a questionnaire annually to screen cancer cases. Cancer diagnoses were then ascertained via medical record review. Deaths were identified by annual mailed questionnaires and periodic linkage to the National Death Index. The primary outcome of interest was the incidence of bladder cancer. In this analysis, bladder cancer case was defined as primary carcinoma of the urinary bladder (International Classification of Diseases for Oncology, Second Edition, codes C67.0–C67.9).

### Statistical Analysis

The carrot, α-carotene and β-carotene intakes were firstly examined as quintiles. Total energy intake was adjusted using the residual method (24). A multivariate Cox proportional hazards model was used to estimate hazard ratios (HRs) and 95% confidence intervals (CIs). Models were established to adjust for covariates of known or suspected risk factors for bladder cancer, including age at trial entry (categorical), sex (male vs. female), race (White, Non-Hispanic vs. Other), body mass index (BMI, < 25 kg/m^2^ vs. ≥ 25 kg/m^2^), education (≤ high school vs. ≥ some college), smoking status (never vs. former ≤ 15 years since quit vs. former > 15 years since quit vs. former year since quit unknown vs. current smoker ≤ 1 pack per day vs. current smoker >1 pack per day vs. current smoker intensity unknown), drinking status (never vs. former vs. current), randomization arm (intervention vs. control), family history of any cancer (yes vs. no), marital status (married vs. not married), Supplemental Beta-Carotene (continuous), Supplemental Calcium (continuous), Supplemental Vitamin A (continuous), Supplemental Vitamin C (continuous), Supplemental Vitamin D (continuous), and Supplemental Vitamin E (continuous). Tests of multiplicative interaction were performed using likelihood-ratio tests compared models with and without the interaction term. The proportional hazards (PH) assumption was examined using the Schoenfeld residual test (25). Restricted cubic spline models (26) were fitted with three knots (i.e., 10th, 50th, and 90th percentiles) to determine the dose-response trend in the association between carrot intake (as a continuous variable) and bladder cancer risk after full adjustment.

### Meta-Analysis

A literature search and selection were performed with PubMed through February 2021 with the following inclusion criteria: (i) assessed the association between carrot intake and bladder cancer risk, (ii) risk estimates with their 95 % CIs were given or sufficient data were reported for calculation, and (iii) the study design was prospective, such as cohort, nested case-control, case-cohort and clinical trial. Two previously published prospective studies (18, 19) and the PLCO cohort were included in the final meta-analysis. If an individual study reported results for different exposures (e.g., raw and cooked carrots) but did not give the overall results, we combined the corresponding risk estimates using the methods proposed by Hamling et al. (27). A DerSimonian and Laird random-effects model (28) was used to calculate summary relative risk (RR) and 95% CI. Heterogeneity among studies was assessed by Q statistic and the I^2^ score (29). All statistical analyses were performed using the software STATA version 15 (Stata Corp, College Station, TX, USA). All tests were two-sided.

## Results

After a median of 12.5 years of follow-up, 762 incident bladder cancer cases occurred. Carrot from diet ranged from 0 to 205.3 g/day, with a median value of 4.31 g/day. Compared to participants who had the highest carrot intake (i.e., quintile 5), participants with the lowest carrot intake (i.e., quintile 1), were younger, had higher BMI, were less likely to be non-Hispanic white, married and current drinkers, were less likely to have a family history of cancer, consumed less total energy and supplements, were more likely to be male and current smokers, and were more likely to have below-college education level (Table 1).

**Table 1:** Main characteristic of participants in the PLCO cancer screening trial by carrot intake.

**Variables**	**Q1 (*n* = 20945)**	**Q2 (*n* = 18997)**	**Q3 (*n* = 22183)**	**Q4 (*n* = 18881)**	**Q5 (*n* = 18644)**	***p*-value**
Age (y), mean (SD)	62.1 (5.2)	62.5 (5.3)	62.2 (5.2)	63.0 (5.4)	62.3 (5.3)	<0.001
Female (*n*, %)	9,940 (47.5%)	8,190 (43.1%)	10,782 (48.6%)	12,116 (64.2%)	10,329 (55.4%)	<0.001
**Arm (*****n*****, %)**						0.82
Screen	10,621 (50.7%)	9,689 (51.0%)	11,366 (51.2%)	9,599 (50.8%)	9,466 (50.8%)	
Control	10,324 (49.3%)	9,308 (49.0%)	10,817 (48.8%)	9,282 (49.2%)	9,178 (49.2%)	
**Smoking (*****n*****, %)**						<0.001
Never	8,443 (40.3%)	8,535 (44.9%)	10,137 (45.7%)	10,626 (56.3%)	9,959 (53.4%)	
Current	3,075 (14.7%)	1,848 (9.7%)	2,107 (9.5%)	1,047 (5.5%)	1,019 (5.5%)	
Former	9,424 (45.0%)	8,612 (45.3%)	9,935 (44.8%)	7,206 (38.2%)	7,664 (41.1%)	
**Education (*****n*****, %)**						<0.001
≤High school	10,059 (48.0%)	8,048 (42.4%)	9,738 (43.9%)	7,304 (38.7%)	6,692 (35.9%)	
≥Some college	10,843 (51.8%)	10,909 (57.4%)	12,405 (55.9%)	11,543 (61.1%)	11,920 (63.9%)	
**BMI (*****n*****, %)**						<0.001
<25.0 kg/m^2^	6,518 (31.1%)	5,958 (31.4%)	6,913 (31.2%)	7,137 (37.8%)	6,631 (35.6%)	
≥25.0 kg/m^2^	14,127 (67.4%)	12,806 (67.4%)	14,967 (67.5%)	11,504 (60.9%)	11,786 (63.2%)	
**Race (*****n*****, %)**						<0.001
White, Non-hispanic	17,781 (84.9%)	17,269 (90.9%)	20,485 (92.3%)	17,546 (92.9%)	17,660 (94.7%)	
Other	3,150 (15.0%)	1,721 (9.1%)	1,693 (7.6%)	1,330 (7.0%)	980 (5.3%)	
**Drinking (*****n*****, %)**						<0.001
Never	1,907 (9.1%)	1,684 (8.9%)	1,972 (8.9%)	2,312 (12.2%)	2,044 (11.0%)	
Former	3,641 (17.4%)	2,760 (14.5%)	3,152 (14.2%)	2,368 (12.5%)	2,481 (13.3%)	
Current	14,683 (70.1%)	14,052 (74.0%)	16,529 (74.5%)	13,650 (72.3%)	13,628 (73.1%)	
Total energy intake (kcal/d), mean (SD)	1570.1 (661.3)	1664.6 (649.4)	1731.1 (660.4)	1747.9 (634.4)	1903.0 (679.4)	<0.001
**Marital status (*****n*****, %)**						<0.001
Married	15,279 (72.9%)	15,140 (79.7%)	17,699 (79.8%)	15,125 (80.1%)	14,888 (79.9%)	
Not married	5,624 (26.9%)	3,819 (20.1%)	4,445 (20.0%)	3,723 (19.7%)	3,729 (20.0%)	
**Family history of cancer (*****n*****, %)**						<0.001
No	9,504 (45.5%)	8,526 (45.0%)	9,674 (43.7%)	8,024 (42.6%)	7,929 (42.6%)	
Yes	11,370 (54.5%)	10,420 (55.0%)	12,445 (56.3%)	10,815 (57.4%)	10,665 (57.4%)	
Supplemental Beta-Carotene (mcg/day), mean (SD)	161.8 (338.6)	173.6 (360.3)	179.4 (368.0)	202.4 (386.2)	208.6 (395.3)	<0.001
Supplemental Calcium (mg/day), mean (SD)	210.8 (333.1)	216.3 (332.4)	244.3 (346.9)	320.0 (373.1)	306.8 (373.5)	<0.001
Supplemental Vitamin A (i.u./day), mean (SD)	2825.0 (3245.7)	2903.4 (3276.3)	2961.6 (3259.2)	3264.6 (3232.1)	3370.4 (3450.3)	<0.001
Supplemental Vitamin C (mg/day), mean (SD)	210.7 (354.9)	220.5 (356.6)	229.7 (360.9)	260.4 (377.8)	279.5 (399.5)	<0.001
Supplemental Vitamin D (mcg/day), mean (SD)	4.8 (4.8)	5.0 (4.8)	5.1 (4.8)	5.6 (4.7)	5.7 (4.7)	<0.001
Supplemental Vitamin E (mg/day), mean (SD)	127.6 (176.4)	133.1 (171.8)	140.8 (175.4)	155.6 (172.5)	164.3 (180.7)	<0.001

*PLCO, prostate, lung, colorectal and ovarian; y, year; SD, standard deviation; BMI, body mass index*.

We found no statistically significant association between dietary carrot intake and the risk of bladder cancer (Table 2). The multivariate-adjusted HR of bladder cancer for participants in the highest category of total carrot intake compared with those in the lowest category was 0.96 (95% CI: 0.76–1.22; *P* for trend = 0.436). Corresponding adjusted HR was 0.98 (95% CI 0.90–1.06) per 1 SD increment of carrot intake. These associations were not modified by smoking status and BMI (*P* for interaction > 0.05).

**Table 2:** Association between energy-adjusted intake of carrot/carotene and bladder cancer risk in the PLCO cancer screening trial.

**Variables**	**Cohort (*n*)**	**Cases (*n*)**	**Crude HR (95% CI), *p*-value**	Adjusted HR (95% CI)^*^, *p*-value
**Carrot**				
Quintile 1	19,930	197	Reference	Reference
Quintile 2	19,930	158	0.79 (0.64–0.97), *p* = 0.025	1.02 (0.83–1.27), *p* = 0.821
Quintile 3	19,930	166	0.82 (0.66–1.00), *p* = 0.054	1.21 (0.98–1.50), *p* = 0.071
Quintile 4	19,930	129	0.62 (0.50–0.78), *p* < 0.001	0.98 (0.78–1.22), *p* = 0.830
Quintile 5	19,930	112	0.54 (0.43–0.68), *p* < 0.001	0.96 (0.76–1.22), *p* = 0.755
			*p* for trend < 0.001	*p* for trend = 0.436
**α-carotene**				
Quintile 1	19,930	209	Reference	Reference
Quintile 2	19,930	171	0.81 (0.66–0.99), *p* = 0.038	1.05 (0.86–1.29), *p* = 0.631
Quintile 3	19,930	135	0.63 (0.51–0.78), *p* < 0.001	0.93 (0.75–1.16), *p* = 0.512
Quintile 4	19,930	130	0.60 (0.48–0.75), *p* < 0.001	0.95 (0.76–1.19), *p* = 0.682
Quintile 5	19,930	117	0.54 (0.43–0.67), *p* < 0.001	0.92 (0.73–1.16), *p* = 0.479
			*p* for trend < 0.001	*p* for trend = 0.369
**β-carotene**				
Quintile 1	19,930	208	Reference	Reference
Quintile 2	19,930	182	0.86 (0.71–1.05), *p* = 0.144	1.15 (0.94–1.41), *p* = 0.171
Quintile 3	19,930	135	0.63 (0.51–0.79), *p* < 0.001	1.04 (0.83–1.30), *p* = 0.747
Quintile 4	19,930	127	0.60 (0.48–0.74), *p* < 0.001	1.04 (0.83–1.31), *p* = 0.723
Quintile 5	19,930	110	0.51 (0.41–0.65), *p* < 0.001	0.97 (0.76–1.23), *p* = 0.807
			*p* for trend < 0.001	*p* for trend = 0.544

*PLCO, prostate, lung, colorectal and ovarian; HR, hazard ratio; CI, confidence interval*.

* *Adjusted for age (categorical), sex (male vs. female), race (White, Non-hispanic vs. Other), body mass index (<25 kg/m^2^ vs. ≥25 kg/m^2^), education (≤ high school vs. ≥some college), smoking status (never vs. former ≤ 15 years since quit vs. former > 15 years since quit vs. former year since quit unknown vs. current smoker ≤ 1 pack per day vs. current smoker >1 pack per day vs. current smoker intensity unknown), alcohol drinking status (never vs. former vs. current), randomization arm (intervention vs. control), family history of any cancer (yes vs. no), marital status (married vs. not married), Supplemental Beta-Carotene (continuous), Supplemental Calcium (continuous), Supplemental Vitamin A (continuous), Supplemental Vitamin C (continuous), Supplemental Vitamin D (continuous), and Supplemental Vitamin E (continuous)*.

**Figure 1:**
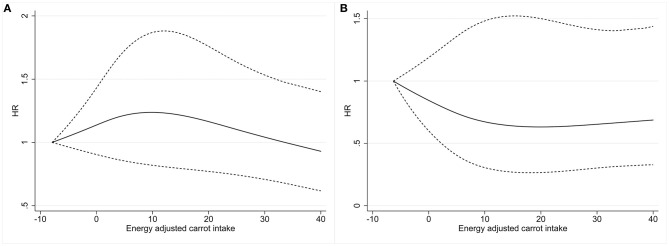
Dose-response analysis was performed using restricted cubic spline model for the association between energy-adjusted dietary carrot intake and bladder cancer risk in males **(A)** and females **(B)**. Solid lines represent point estimates and dashed lines represent 95% CIs. HRs were calculated by restricted cubic spline regression with three knots (i.e., 10th, 50th, and 90th percentiles) adjusting for age, sex, race, BMI, education, smoking status, drinking status, marital status, family history of cancer, arm, Supplemental Beta-Carotene, Supplemental Calcium, Supplemental Vitamin A, Supplemental Vitamin C, Supplemental Vitamin D, and Supplemental Vitamin E. HR, hazard ratio; CI, confidence interval; BMI, body mass index.

A spline regression plot of bladder cancer risk in relation to carrot intake has been shown in Figure 1, which suggested that consumption of carrot was not associated with bladder cancer incidence. There was no statistical evidence for nonlinearity (*P* for nonlinearity > 0.05). Similar results were obtained when excluding cases diagnosed within the first two years of follow-up (Fully adjusted model: HR_*Q*5__*vs*.__*Q*1_ = 0.96, 95% CI 0.76–1.23), when excluding participants with family history (Fully adjusted model: HR_*Q*5__*vs*.__*Q*1_ = 0.83, 95% CI 0.57–1.20), and when further adjusted for hypertension (Fully adjusted model: HR_*Q*5__*vs*.__*Q*1_ = 0.95, 95% CI 0.75–1.21).

α-carotene and β-carotene are found in high amounts in carrots. There were no statistically significant associations between consumption of α-carotene (Fully adjusted model: HR_*Q*5__*vs*.__*Q*1_ = 0.92, 95% CI 0.73–1.16; *P* for trend = 0.369) or β-carotene (Fully adjusted model: HR_*Q*5__*vs*.__*Q*1_ = 0.97, 95% CI 0.76–1.23; *P* for trend = 0.544) and bladder cancer risk (Table 2).

We also performed a meta-analysis based on two previous cohort studies and our study. As a result, no significant association was found for carrot intake and bladder cancer risk (Summary HR 1.02, 95% CI 0.95–1.10) without obvious heterogeneity between studies (*P* for heterogeneity = 0.859, *I*^2^ = 0.0%).

## Discussion

In this large prospective PLCO cohort and meta-analysis of cohort studies, no statistically significant association between carrot intake and bladder cancer risk was observed. Similar results were obtained when excluding cases diagnosed within the first two years of follow-up.

Although three case-control studies (14–16) have provided some evidence of a potential inverse association between carrot intake and bladder cancer risk, these studies may be prone to selection and recall biases. Our results, which were based on a prospective cohort study, were comparable to the findings reported by Sakauchi et al. in 2005 (18). They investigated the association between dietary habits and risk of urothelial cancer in the JACC Study and found that higher carrot intake was not associated with the risk of urothelial cancer. By contrast, Zeegers et al. (19) reported that there was a statistically significant inverse association between cooked carrots intake and urothelial cancer risk in the Netherlands Cohort Study. The inconsistent results between the previous studies and the present study may be caused by the differences in sample size, method of exposure assessment, and the adjusted confounders.

Carrot consumption has been inversely associated with various health outcomes, including colorectal cancer (30), lung cancer (31), prostate cancer (32), breast cancer (33), and stroke mortality (34). However, in our study, we failed to find a significant association between carrot intake and bladder cancer risk. In addition, we also investigated the potential effect of α-carotene and β-carotene on bladder cancer and found that neither of them was associated with the bladder cancer risk. These findings were consistent with the results of a recent meta-analysis based on eligible observational studies, which reported that dietary intakes of α-Carotene and β-carotene were not statistically significantly associated with bladder cancer risk (35).

The strengths of the current study included the prospective design, large population size, a comprehensive list of potential confounders, and long duration of follow-up, which substantially decreased the chance of reverse causality and provided substantial power to detect differences in bladder cancer incidence if they truly existed. The broad ranges of dietary carrot intake allowed us to comprehensively evaluate the effects of carrot at different intake levels.

This study had several limitations. First, despite full adjustment for established and suspected confounders, we could not exclude the possibility of residual or unmeasured confounding. Second, the vast majority of participants included in this study were non-Hispanic Whites, which may limit its generalizability to other populations. Third, only a single measurement for dietary intake was performed at baseline and thus we were not able to take into account diet change over time. Finally, PLCO study did not provide data for raw and cooked carrot separately, which made it impossible to evaluate their effect on bladder cancer risk.

In summary, analysis of the PLCO cohort did not provide evidence that dietary consumption of carrot, α-carotene or β-carotene was associated with the risk of bladder cancer.

## Data Availability Statement

The datasets presented in this study can be found in online repositories. The names of the repository/repositories and accession number(s) can be found below: https://cdas.cancer.gov/datasets/plco/.

## Ethics Statement

The studies involving human participants were reviewed and approved by National Cancer Institute. The patients/participants provided their written informed consent to participate in this study.

## Author Contributions

XX and DX contributed to the conception or design of the work. XX, YZ, and SY contributed to the acquisition, analysis, or interpretation of data for the work. XX and SL drafted the manuscript. BX, HM, and SW critically revised the manuscript. All authors gave final approval and agree to be accountable for all aspects of work ensuring integrity and accuracy.

## Author Disclaimer

The statements contained herein are solely those of the authors and do not represent or imply concurrence or endorsement by NCI.

## Conflict of Interest

The authors declare that the research was conducted in the absence of any commercial or financial relationships that could be construed as a potential conflict of interest.

## Publisher's Note

All claims expressed in this article are solely those of the authors and do not necessarily represent those of their affiliated organizations, or those of the publisher, the editors and the reviewers. Any product that may be evaluated in this article, or claim that may be made by its manufacturer, is not guaranteed or endorsed by the publisher.

## References

[1] Antoni S Ferlay J Soerjomataram I Znaor A Jemal A Bray F. Bladder cancer incidence and mortality: a global overview and recent trends. Eur Urol. (2017) 71:96–108. 10.1016/j.eururo.2016.06.010 27370177

[2] Cumberbatch MGK Jubber I Black PC Esperto F Figueroa JD Kamat AM. Epidemiology of bladder cancer: a systematic review and contemporary update of risk factors in 2018. Eur Urol. (2018) 74:784–95. 10.1016/j.eururo.2018.09.001 30268659

[3] Mertens LS Neuzillet Y Horenblas S van Rhijn BWG. Landmarks in non-muscle-invasive bladder cancer. Nat Rev Urol. (2014) 11:476–80. 10.1038/nrurol.2014.130 24980189

[4] Scarpato KR Morgans AK Moses KA. Optimal management of muscle-invasive bladder cancer - a review. Res Rep Urol. (2015) 7:143–51. 10.2147/RRU.S73566 26380230 PMC4567228

[5] Cumberbatch MG Rota M Catto JW La Vecchia C. The role of tobacco smoke in bladder and kidney carcinogenesis: a comparison of exposures and meta-analysis of incidence and mortality risks. Eur Urol. (2016) 70:458–66. 10.1016/j.eururo.2015.06.042 26149669

[6] Westhoff E Maria de Oliveira-Neumayer J Aben KK Vrieling A Kiemeney LA. Low awareness of risk factors among bladder cancer survivors: new evidence and a literature overview. Eur J Cancer. (2016) 60:136–45. 10.1016/j.ejca.2016.03.071 27125965

[7] Keimling M Behrens G Schmid D Jochem C Leitzmann MF. The association between physical activity and bladder cancer: systematic review and meta-analysis. Br J Cancer. (2014) 110:1862–70. 10.1038/bjc.2014.77 24594995 PMC3974090

[8] Xu X. Processed meat intake and bladder cancer risk in the prostate, lung, colorectal, and ovarian (PLCO) cohort. Cancer Epidemiol Biomarkers Prev. (2019) 28:1993–7. 10.1158/1055-9965.EPI-19-0604 31533945

[9] Golabek T Bukowczan J Sobczynski R Leszczyszyn J Chlosta PL. The role of micronutrients in the risk of urinary tract cancer. Arch Med Sci. (2016) 12:436–47. 10.5114/aoms.2016.59271 27186192 PMC4848374

[10] Frei B. Reactive oxygen species and antioxidant vitamins: mechanisms of action. Am J Med. (1994) 97:5S–13S; discussion 22S−8S. 10.1016/0002-9343(94)90292-5 8085584

[11] Shin J Song MH Oh JW Keum YS Saini RK. Pro-oxidant actions of carotenoids in triggering apoptosis of cancer cells: a review of emerging evidence. Antioxidants. (2020) 9:532. 10.3390/antiox9060532 32560478 PMC7346220

[12] Ngoc NB Lv P Zhao WE. Suppressive effects of lycopene and β-carotene on the viability of the human esophageal squamous carcinoma cell line EC109. Oncol Lett. (2018) 15:6727–32. 10.3892/ol.2018.8175 29731858 PMC5920922

[13] Cui Y Lu Z Bai L Shi Z Zhao WE Zhao B. beta-Carotene induces apoptosis and up-regulates peroxisome proliferator-activated receptor gamma expression and reactive oxygen species production in MCF-7 cancer cells. Eur J Cancer. (2007) 43:2590–601. 10.1016/j.ejca.2007.08.015 17911009

[14] Mettlin C Graham S. Dietary risk factors in human bladder cancer. Am J Epidemiol. (1979) 110:255–63. 10.1093/oxfordjournals.aje.a112810 582494

[15] Pohlabeln H Jöckel KH Bolm-Audorff U. Non-occupational risk factors for cancer of the lower urinary tract in Germany. Eur J Epidemiol. (1999) 15:411–9. 10.1023/A:1007595809278 10442466

[16] Radosavljević V Janković S Marinković J Dokić M. Diet and bladder cancer: a case-control study. Int Urol Nephrol. (2005) 37:283–9. 10.1007/s11255-004-4710-8 16142557

[17] Luo X Lu H Li Y Wang S. Carrot intake and incidence of urothelial cancer: a systematic review and meta-analysis. Oncotarget. (2017) 8:77957–62. 10.18632/oncotarget.19832 29100438 PMC5652827

[18] Sakauchi F Mori M Washio M Watanabe Y Ozasa K Hayashi K. Dietary habits and risk of urothelial cancer incidence in the JACC study. J Epidemiol. (2005) 15(Suppl. 2):S190–5. 10.2188/jea.15.S190 16127233 PMC8639034

[19] Zeegers MP Goldbohm RA van den Brandt PA. Consumption of vegetables and fruits and urothelial cancer incidence: a prospective study. Cancer Epidemiol Biomarkers Prev. (2001) 10:1121–8. 11700259

[20] Prorok PC Andriole GL Bresalier RS Buys SS Chia D Crawford ED. Design of the prostate, lung, colorectal and ovarian (PLCO) cancer screening trial. Control Clin Trials. (2000) 21(Suppl. 6):273S–309S. 10.1016/S0197-2456(00)00098-2 11189684

[21] Gren L Broski K Childs J Cordes J Engelhard D Gahagan B. Recruitment methods employed in the prostate, lung, colorectal, and ovarian cancer screening trial. Clin Trials. (2009) 6:52–9. 10.1177/1740774508100974 19254935 PMC4651181

[22] Subar AF Thompson FE Kipnis V Midthune D Hurwitz P McNutt S. Comparative validation of the block, willett, and national cancer institute food frequency questionnaires : the eating at America's table study. Am J Epidemiol. (2001) 154:1089–99. 10.1093/aje/154.12.1089 11744511

[23] Subar AF Midthune D Kulldorff M Brown CC Thompson FE Kipnis V. Evaluation of alternative approaches to assign nutrient values to food groups in food frequency questionnaires. Am J Epidemiol. (2000) 152:279–86. 10.1093/aje/152.3.279 10933275

[24] Willett WC Howe GR Kushi LH. Adjustment for total energy intake in epidemiologic studies. Am J Clin Nutr. (1997) 65(Suppl. 4):1220S–8S; discussion 9S−31S. 10.1093/ajcn/65.4.1220S 9094926

[25] Schoenfeld D. Chi-squared goodness-of-fit tests for the proportional hazards regression model. Biometrika. (1980) 67:145–53. 10.1093/biomet/67.1.145

[26] Marrie RA Dawson NV Garland A. Quantile regression and restricted cubic splines are useful for exploring relationships between continuous variables. J Clin Epidemiol. (2009) 62:511–7 e1. 10.1016/j.jclinepi.2008.05.015 19135859

[27] Hamling J Lee P Weitkunat R Ambühl M. Facilitating meta-analyses by deriving relative effect and precision estimates for alternative comparisons from a set of estimates presented by exposure level or disease category. Stat Med. (2008) 27:954–70. 10.1002/sim.3013 17676579

[28] DerSimonian R Laird N. Meta-analysis in clinical trials. Control Clin Trials. (1986) 7:177–88. 10.1016/0197-2456(86)90046-2 3802833

[29] Higgins JP Thompson SG. Quantifying heterogeneity in a meta-analysis. Stat Med. (2002) 21:1539–58. 10.1002/sim.1186 12111919

[30] Deding U Baatrup G Christensen LP Kobaek-Larsen M. Carrot intake and risk of colorectal cancer: a prospective cohort study of 57,053 danes. Nutrients. (2020) 12:332. 10.3390/nu12020332 32012660 PMC7071341

[31] Xu H Jiang H Yang W Song F Yan S Wang C. Is carrot consumption associated with a decreased risk of lung cancer? A meta-analysis of observational studies. Br J Nutr. (2019) 122:488–98. 10.1017/S0007114519001107 31552816

[32] Xu X Cheng Y Li S Zhu Y Zheng X Mao Q. Dietary carrot consumption and the risk of prostate cancer. Eur J Nutr. (2014) 53:1615–23. 10.1007/s00394-014-0667-2 24519559

[33] Chen H Shao F Zhang F Miao Q. Association between dietary carrot intake and breast cancer: a meta-analysis. Medicine. (2018) 97:e12164. 10.1097/MD.0000000000012164 30212943 PMC6156046

[34] Zurbau A Au-Yeung F Blanco Mejia S Khan TA Vuksan V Jovanovski E. Relation of different fruit and vegetable sources with incident cardiovascular outcomes: a systematic review and meta-analysis of prospective cohort studies. J Am Heart Assoc. (2020) 9:e017728. 10.1161/JAHA.120.017728 33000670 PMC7792377

[35] Wu S Liu Y Michalek JE Mesa RA Parma DL Rodriguez R. Carotenoid intake and circulating carotenoids are inversely associated with the risk of bladder cancer: a dose-response meta-analysis. Adv Nutr. (2020) 11:630–43. 10.1093/advances/nmz120 31800007 PMC7231589

